# A Comparative Analysis of Glomerulus Development in the Pronephros of Medaka and Zebrafish

**DOI:** 10.1371/journal.pone.0045286

**Published:** 2012-09-18

**Authors:** Koichiro Ichimura, Ekaterina Bubenshchikova, Rebecca Powell, Yayoi Fukuyo, Tomomi Nakamura, Uyen Tran, Shoji Oda, Minoru Tanaka, Oliver Wessely, Hidetake Kurihara, Tatsuo Sakai, Tomoko Obara

**Affiliations:** 1 Department of Cell Biology, University of Oklahoma Health Science Center, Oklahoma City, Oklahoma, United States of America; 2 Department of Anatomy and Life Structure, Juntendo University School of Medicine, Tokyo, Japan; 3 Department of Biological Science and Technology, Graduate School of Industrial Science and Technology, Tokyo University of Science, Noda, Chiba, Japan; 4 Department of Cell Biology, Lerner Research Institute/Cleveland Clinic Foundation, Cleveland, Ohio, United States of America; 5 Department of Integrated Biosciences, Graduate School of Frontier Sciences, The University of Tokyo, Kashiwa, Chiba, Japan; 6 Laboratory of Molecular Genetics for Reproduction, National Institute for Basic Biology, Aichi, Japan; Leibniz Institute for Age Research - Fritz Lipmann Institute (FLI), Germany

## Abstract

The glomerulus of the vertebrate kidney links the vasculature to the excretory system and produces the primary urine. It is a component of every single nephron in the complex mammalian metanephros and also in the primitive pronephros of fish and amphibian larvae. This systematic work highlights the benefits of using teleost models to understand the pronephric glomerulus development. The morphological processes forming the pronephric glomerulus are astoundingly different between medaka and zebrafish. (1) The glomerular primordium of medaka - unlike the one of zebrafish - exhibits a C-shaped epithelial layer. (2) The C-shaped primordium contains a characteristic balloon-like capillary, which is subsequently divided into several smaller capillaries. (3) In zebrafish, the bilateral pair of pronephric glomeruli is fused at the midline to form a glomerulus, while in medaka the two parts remain unmerged due to the interposition of the interglomerular mesangium. (4) Throughout pronephric development the interglomerular mesangial cells exhibit numerous cytoplasmic granules, which are reminiscent of renin-producing (juxtaglomerular) cells in the mammalian afferent arterioles. Our systematic analysis of medaka and zebrafish demonstrates that in fish, the morphogenesis of the pronephric glomerulus is not stereotypical. These differences need be taken into account in future analyses of medaka mutants with glomerulus defects.

## Introduction

During vertebrate ontogeny and phylogeny, three types of kidney are distinguishable on the basis of their localization and developmental process [1], [2]. The pronephros is the first kidney that forms and functions as a primary osmoregulatory organ in the larvae of teleost fishes and amphibians [3], [4]. Teleost fishes generally possess a pair of functional pronephroi, which consist of three anatomical subunits (glomerulus, pronephric tubule, and pronephric duct) [5]. In vertebrates, the pronephros is the first kidney to form and is succeeded by the mesonephros, which is the adult kidney in fishes and amphibians. In amniotes, the pronephros is formed, but remains non-functional; the mesonephros serves as the embryonic kidney and the metanephros as the adult kidney. The progression to the more advanced kidney form is always accompanied by the degeneration of the previous kind [6].

The glomerulus exhibits a common structural organization regardless of the taxonomic groups of vertebrates and the kidney types, but is obviously adapted to the different developmental and homeostatic requirements [7], [8]. Structurally, the glomerulus can be divided into vascular and epithelial regions. The vascular region is the core structure of the glomerulus and consists of the capillary network and mesangium. The vascular region is surrounded by the epithelial region, a sheet-like structure consisting of the podocytes and glomerular basement membrane (GBM). The vertebrate podocyte is an epithelial cell highly specialized for glomerular filtration. It is composed of three subcellular compartments: the cell body, the primary processes, and the foot processes [9], [10]. Podocytes adhere to the GBM primarily via their numerous foot processes, which are essential to form the size exclusion barrier. The space between adjacent podocyte foot processes is spanned by a slit diaphragm. The cell bodies of podocytes are separated from the GBM via the subpodocyte space [11] and the primary processes connect the foot processes to the cell body. This basic cytoarchitecture of podocytes is highly conserved throughout various kinds of vertebrate [12]–[19].

**Figure 1:**
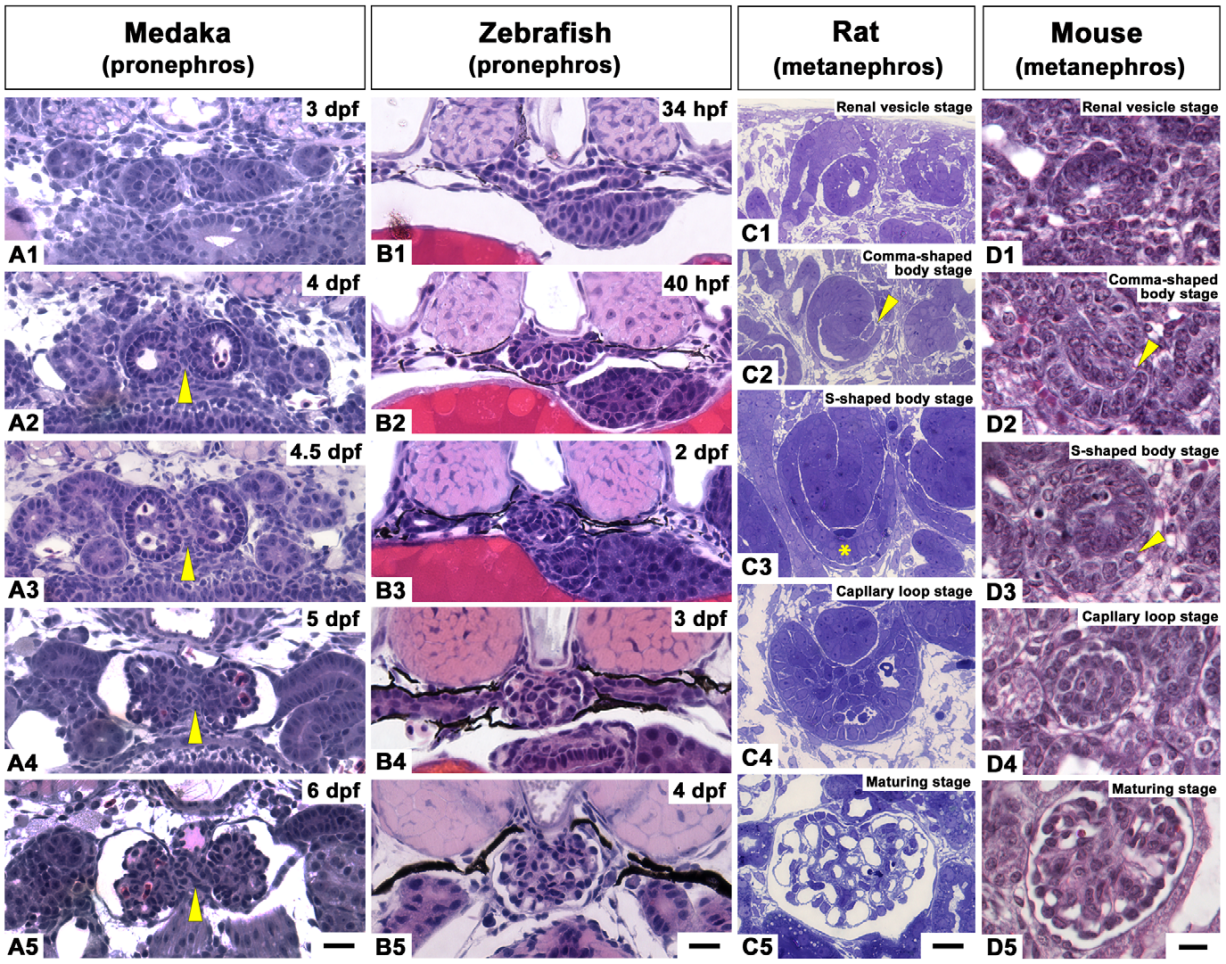
Glomerulus development in medaka and zebrafish pronephros in comparison to mouse and rat metanephros. A1-B5: Pronephric glomerulus development in 3 to 6 dpf medaka (A1-A5) and 34 hpf to 4 dpf zebrafish (B1-B5) by JB4 cross sections stained by hematoxylin and eosin. The glomerular primordium of medaka exhibited a C-shaped epithelial layer (A2, A3), which is similar to the mouse and rat S-shaped body stage (C3, D3), unlike in zebrafish (B2). The C-shaped primordium contained a characteristic balloon-like or sinusoidal capillary. The paired pronephric glomerulus was fused at the midline to form a glomerulus in zebrafish (B3), but remained separated into two parts by the interposition of an interglomerular mesangium in medaka (arrowheads in A2-A5). C1-D5: Metanephric glomerulus development in rat epoxy resin sections stained by toluidine blue (C1-C5) and in mouse E18.5 kidney sections stained with H&E (D1-D5). Cross section of rat and mouse metanephros shows renal vesicle (C1, D1), comma-shaped body (C2, D2), S-shaped body at (C3, D3), capillary loop at (C4, D4), and maturing glomerulus (C5, D5). Rat vascular cleft in C2 (arrowhead) and primitive podocyte layer in C3 (asterisk). Mouse vascular cleft in D2 and D3 (arrowheads). Scale bars = 10 µm.

A number of studies have investigated the morphological process of glomerulogenesis in teleost fishes, amphibians, reptiles, birds, and mammals [5], [20]–[23]. However, the morphological processes are best understood for the metanephric glomerulus of mammals [24], [25], which goes through a series of developmental stages, forming the renal vesicle, comma-shaped body, S-shaped body, capillary loop and maturing glomerulus (Fig. 1C1-5, 1D1-5). The renal vesicle is the primordial structure of the nephron composed of a single cuboidal epithelium. A vascular cleft invaginates at the wall of the renal vesicle to form the comma-shaped body. Endothelial cells and mesenchymal cells invade into the vascular cleft to form the S-shaped body. At this stage, primitive podocytes rearrange into a single columnar epithelium. Glomerular capillaries develop in the vascular cleft and invaginate into the podocyte epithelial layer in the capillary loop stage. The layer then becomes attenuated and gradually reorganizes into the mature glomerular form observed in adults.

In addition to mammalian metanephros, the pronephros of small aquatic animals has been used to study embryonic kidney development [26], [27]. Among those, zebrafish is very popular because of its optical clarity, which is ideal for observing and manipulating organ development, its high fecundity and its rapid development of internal organs. As in other fishes and amphibians, the zebrafish pronephros is the first kidney to form during embryogenesis and is required to maintain proper osmoregulation [5], [27], [28]. Moreover, its organization resembles that of the mammalian nephron [29]. In particular, the pronephric glomerulus is composed of the same cell types as mammalian glomeruli, including the fenestrated endothelial cells of the capillary tufts and the podocytes with their extensive foot processes [5], [30].

In a recent screen, we have identified the first mutants in medaka that affect development of the pronephric glomerulus. For accurate phenotypic analysis of these mutants, it is essential to clearly understand the normal processes of pronephric glomerulus development in medaka, as was the case for zebrafish [5]. However, medaka glomerulus development was previously only characterized by histology and *in situ* hybridization using the podocyte transcription factor *wt1a* [31].

In this study we systematically described the normal developmental process of each glomerular component (podocytes, endothelial cells, mesangial matrix and GBM) in the medaka pronephros and compared them with those found in the zebrafish pronephros and mammalian metanephros (rat and mouse). We believe that such a comparative study of glomerulus development and barrier formation is essential for phenotypic analysis of mutant medaka and may provide invaluable insights to understand human glomerular diseases.

**Figure 2:**
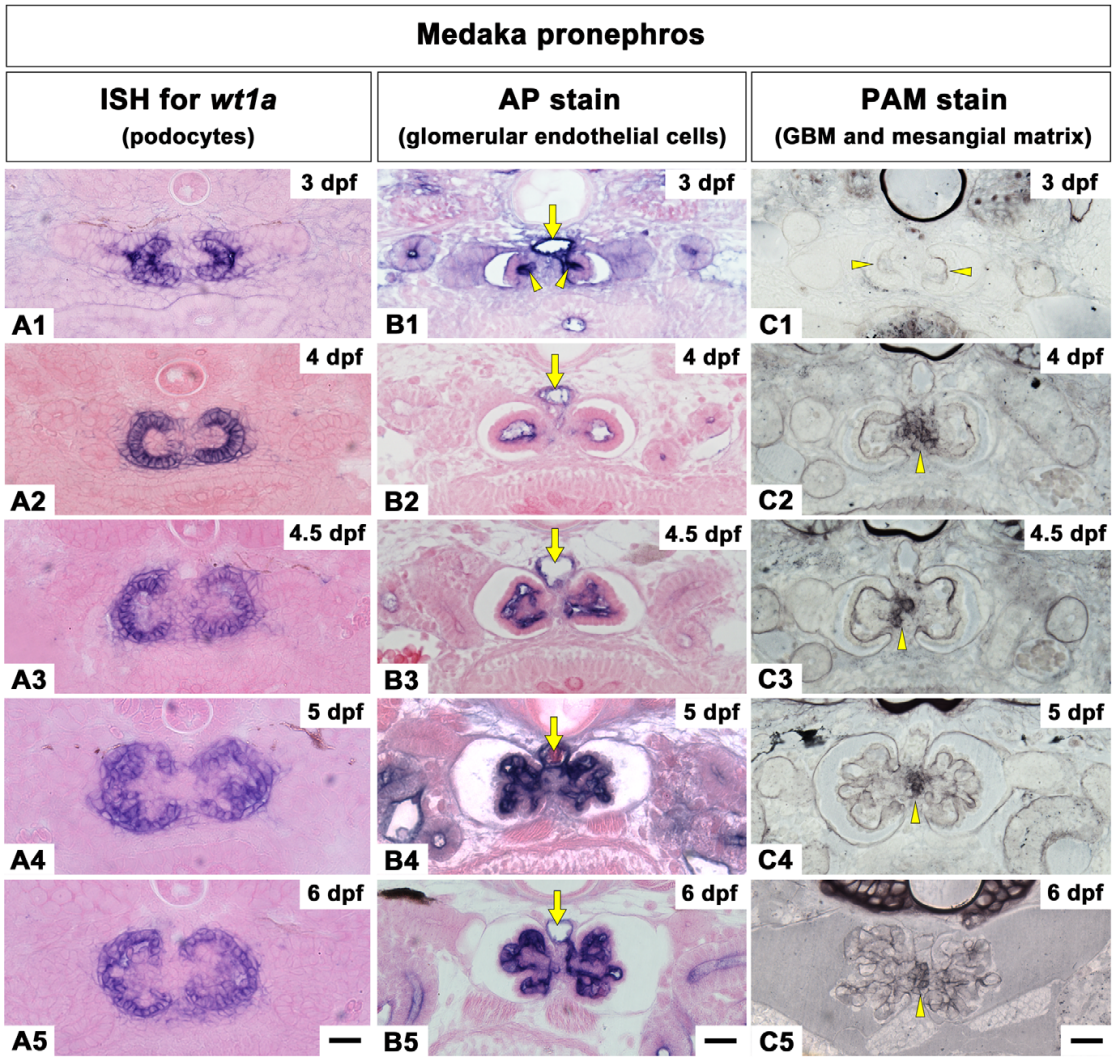
Temporal specification of the three glomerular components during glomerular development in medaka. A1-A5: *wt1a* mRNA is expressed in the forming glomerulus from 3 dpf until 6 dpf. Cross sections show that *wt1a* positive layers of the paired glomerular primordium lie adjacent to each other throughout the pronephric development. B1-B5: Glomerular capillaries are visualized by detecting endogeneous alkaline phosphatase (AP) in endothelial cells. AP-positive endothelial cells are detected in the invaginated portion of nephron primordium from 3 dpf (arrowheads in B1). The balloon-like capillary starts forming at 4 dpf (B2), and then divides into a few smaller capillaries by mesangium by 4.5 dpf (B3). Capillaries continue to grow at 5 dpf (B4) and integrate with the glomerulus by 6 dpf (B5). Dorsal aorta (arrows in B1-B5). C1-C5: Glomerular basement membrane (GBM) and mesangial matrix are detected by periodic acid-methenamine-silver (PAM) stain. The invaginated portion of the basement membrane is densely labeled with PAM stain in comparison with other regions (arrowheads in C1). The PAM-positive matrix is formed at the interglomerular mesangial region (arrowhead in C2-C5) and has an amorphous appearance. Scale bars = 10 µm.

**Figure 3:**
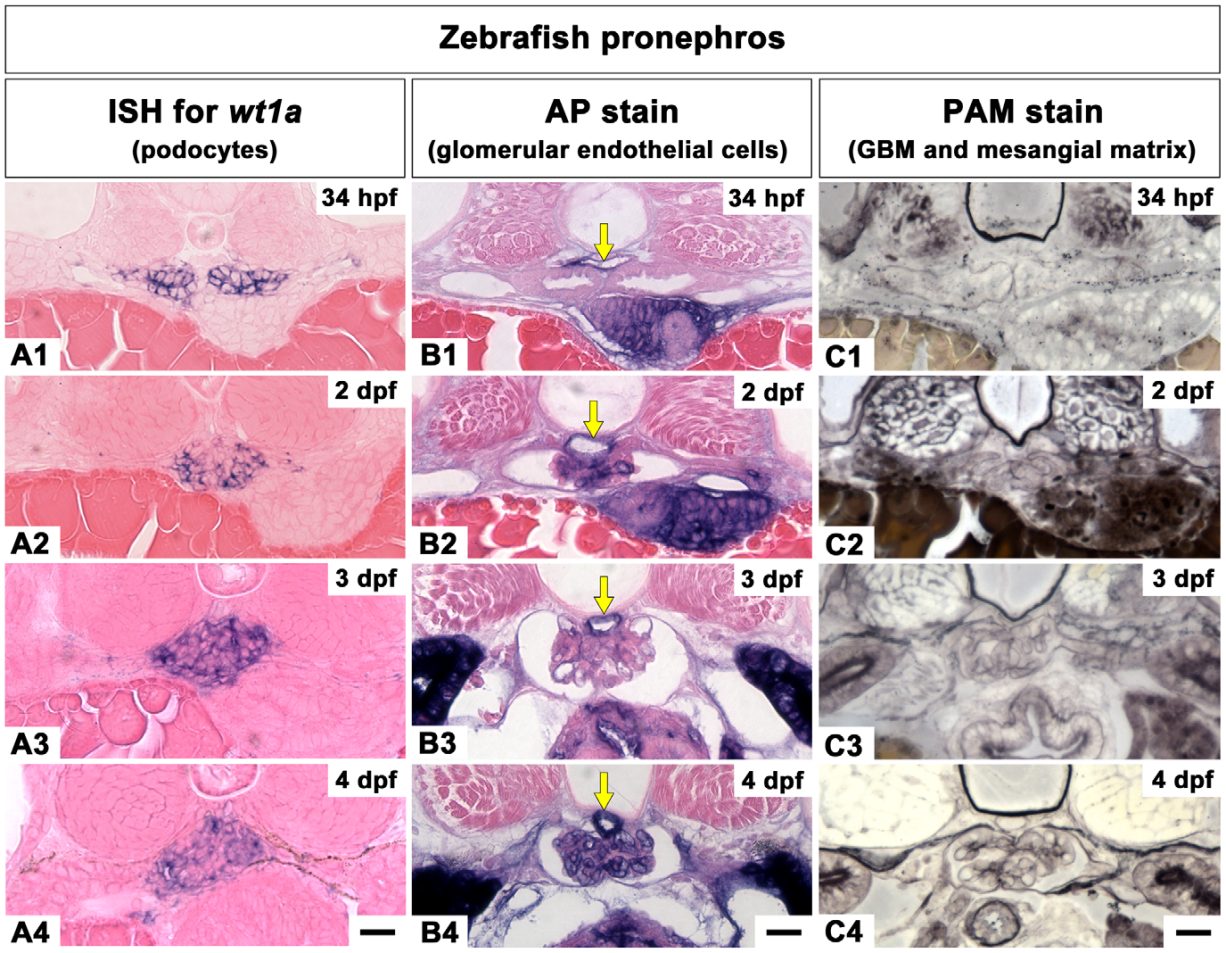
Temporal specification of the three glomerular components during glomerular development in zebrafish. A1-A4: *wt1a* mRNA is expressed in the forming glomerulus from 34 hpf to 4 dpf. Cross sections show *wt1a*-positive podocyte layers of the paired glomeruli at 34 hpf (A1). Unlike medaka, zebrafish *wt1a*-positive podocytes are merged at 2 dpf (A2). B1-B4: Glomerular capillaries are detected by AP stain. At 34 hpf, the flattened nephron primordia show proximity to the dorsal aorta (B1). Glomerular capillaries are found at 2 dpf (B2). Dorsal aorta (arrows in B1-B4). C1-C4: GBM and mesangial matrix are detected by PAM stain. The paired glomeruli fuse at the midline and the interglomerular mesangium is not formed (C2-C4). Scale bars = 10 µm.

## Materials and Methods

### Ethics Statement

All experiments were performed in strict accordance with the recommendation in the Guide for the Care and Use of Laboratory Animals of the National Institutes of Health. The zebrafish and medaka experiments were covered by protocols approved from the Institutional Animal Care and Use Committee of the University of Oklahoma Health Science Center (IACUC protocol #12-033 to TO), the mouse study by a protocol approved from the IACUC committee of the Cleveland Clinic Foundation (IACUC protocol #2011-0478 to OW), and the rat study by a protocol approved from the ethical committee of Juntendo University School of Medicine (protocol #220173 to TS).

### Fish Husbandry

Medaka *Oryzias latipes* (Cab strain) and zebrafish *Danio rerio* (AB strain) were maintained and raised at 28.5°C under a 14-hr light/10-hr dark cycle. Medaka and zebrafish embryos were kept at 28.5°C in medaka embryo culture medium (17 mM NaCl, 0.4 mM KCl, 0.3 mM CaCl_2_, 0.65 mM MgSO_4_, 0.01% methylene blue), and 0.5X E2 egg medium (7.5 mM NaCl, 0.25 mM KCl, 0.5 mM CaCl_2_, 0.5 mM MgSO_4_, 0.075 mM KH_2_PO_4_, 0.025 mM Na_2_HPO_4_, 0.35 mM NaHCO_3_, 0.01% methylene blue), respectively. To suppress pigmentation of zebrafish embryos, 0.0045% 1-Phenyl-2-thiourea (Sigma-Aldrich) was added to 0.5X E2 egg medium as needed. Embryos and larvae were staged according to hours post-fertilization (hpf) or days post-fertilization (dpf) [32]–[34].

### Histological Analysis

Embryos were fixed with histology fixative (1% glutaraldehyde, 1% paraformaldehyde, 3% sucrose in 70 mM phosphate buffer (PB, pH 7.3)) overnight at 4°C, dehydrated by graded series of methanol and embedded in JB4 resin (Polysciences, Inc.). 4 µm sections were cut by a RN2255 microtome (Leica) and stained with Harris hematoxylin and special eosin II (BBC Biochemical). After mounted in Poly-Mount (Polysciences, Inc.), the stained sections were imaged with a Provis AX-70 microscope (Olympus) equipped with a RETIGA EXi digital camera (QImaging). Rat metanephric kidney at embryonic day 18 to postnatal day 2 were fixed with 2.5% glutaraldehyde in 0.1M PB, post-fixed with 0.4% OsO_4_ in 0.1M PB, dehyrdrated with graded series of acetone, and embedded in Epoxy resin. 0.5 µm sections were stained using Toluidine blue. Mouse metanephric kidneys at embryonic day 18.5 were fixed with Bouin’s fixative, washed in 70% ethanol, and embedded in Paraplast (Structure Probe, Inc.). 5 µm sections were stained using hematoxylin and eosin.

**Figure 4:**
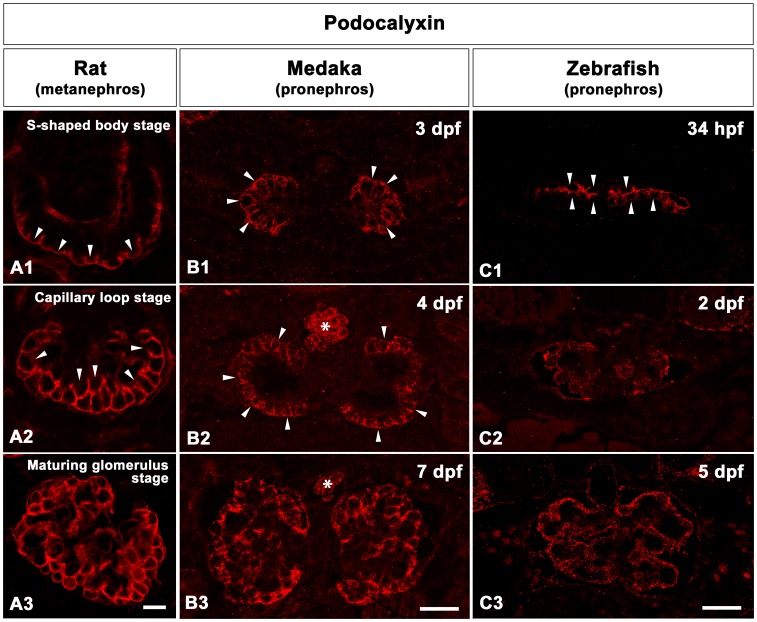
The progression of podocyte differentiation visualized by Podocalyxin immunostaining. A1-A3: Cross sections of rat metanephric glomeruli. Throughout the rat metanephric glomeruli development, Podocalyxin is mainly localized at the apical membrane of podocytes. At the S-shaped body stage, Podocalyxin marks the apical membrane of the individual podocytes in a cap-shaped pattern (arrowheads in A1). By early capillary loop stage, Podocalyxin localizes in a U-shaped pattern in the individual podocytes (arrowheads A2). By the maturing glomerulus stage, Podocalyxin staining is detected along the entire surface of podocytes (A3). B1-B3: Cross sections of medaka pronephric glomeruli. Podocalyxin immunostaining is detected in the individual podocytes as a U-shaped pattern at 3 and 4 dpf (B1, B2). At 7 dpf, Podocalyxin staining is detected along the entire surface of podocytes (B3). Asterisks indicate blood cells in dorsal aorta. C1-C3: Cross sections of zebrafish pronephric glomeruli. At 34 hpf, Podocalyxin marks the apical membrane of individual podocytes at 34 hpf (C1). By 2 dpf (C2) and 5 dpf (C3), Podocalyxin immunostaining is expressed along entire surface of podocytes. Scale bars = 10 µm.

**Figure 5:**
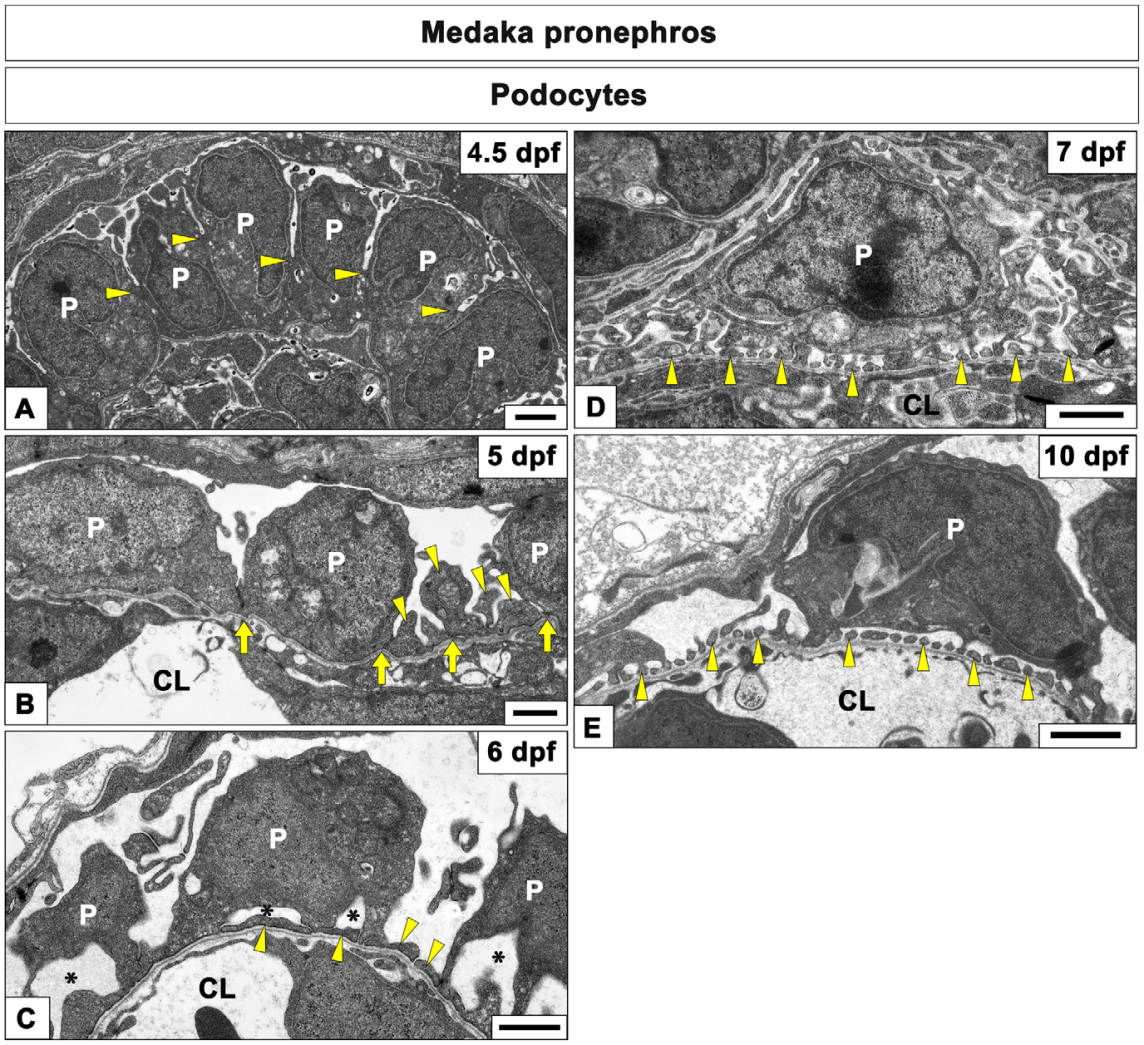
Transmission electron microscopy of podocytes development in medaka pronephric glomerulus. A-E: The ultrastructure of pronephric glomerulus cross sections. A: At 4.5 dpf, podocytes form a single columnar epithelium and are connected via intercellular junctions (arrowheads), which are located in the middle of the cell height. B: The intercellular junctions of podocytes are located in the vicinity of the GBM (arrows) at 5 dpf. Podocytes interdigitate at the cell periphery to form irregularly-shaped processes (arrowheads). C: The podocyte cell body is detached from the GBM to form a subpodocyte space (asterisks) by 6 dpf. The irregularly-shaped processes become flattened (arrowheads); however, foot processes with silt diaphragms have not formed yet. D: Foot processes connected by slit diaphragm are formed in some capillary walls (arrowheads) at 7 dpf. E: Foot processes are found in the most capillary walls (arrowheads) at 10 dpf. CL, capillary lumen; P, podocyte cell body: PE. Scale bars = 1 µm.

### *In situ* Hybridization

Medaka partial-length *wt1a* and *renin* cDNA were obtained by RT-PCR from total RNA isolated from 5–7 dpf Cab embryos using RNAqueous-4PCR Kit (Ambion). RT-PCR was performed using the SuperScript III One-Step RT-PCR System with Platinum Taq High Fidelity (Invitrogen) followed by a second PCR using Phusion High-Fidelity DNA Polymerase (New England BioLabs). The primers used for RT-PCR were: *zfwt1a*-56F1: 5′-CCG GTG GAA ACG GTA ACT GTA-3′, *zfwt1a*-1161R1: 5′-TCT GCA GTT GAA GGG CTT CTC-3′, *mewtla*-126F1: 5′-TGC TGC CTC ACC TTA CTC CTC-3′, *mewt1a*-1039R1: 5′-CGC ATT CGA ACG GTT TAA CTC-3′, *merenin*-31F1: 5′- TCT CCA GCC CAG ATG TTC AAT-3′, *merenin*-959R1: 5′- GCT GCA GCA AAT CCT ATC CTG-3′. The primers used for 2nd PCR were, *zfwt1a*-240F2: 5′-GCA CTT CTC CGG ACA GTT CAC-3′, *zfwt1a*-1004R2T7: 5′-GGT AAT ACG ACT CAC TAT AGG GAG AAC CTG CGA CCA CAG TCT-3′, *mewt1a*-258F2: 5′-CTT CTC GGG ACA GTT CAC AGG-3′, *mewtla*-992R2T7: 5′-GGT AAT ACG ACT CAC TAT AGG AGC TGG TCA GAG CGT GAA AAG-3′, *merenin*-105F2: 5′-ACC CTT TTC CAC TGC CTG TTT-3′, *merenin*-850R2T7: 5′-GGT AAT ACG ACT CAC TAT AGG GAC CCC TGA AAG TGA CTG TGC-3′. The 2nd PCR product was used as a template for digoxigenin-labeled anti-sense RNA probe. All probes were synthesized using T7 RNA polymerase (New England BioLabs) and DIG-RNA labeling (Roche) according to the manufacturer’s instructions. Embryos were fixed in 4% PFA, 0.1% Tween 20 in PBS for 2 h at RT and changed to 100% MeOH and stored at −20°C. Whole mount *in situ* hybridization was performed as described previously [35]. Alkaline phosphatase-conjugated anti-digoxigenin (Roche) was used to localize the probes. NBT/BCIP (Roche) was used as the chromogenic substrate to produce the blue staining. After color development, samples were dehydrated with graded series of methanol and embedded in JB4 resin (Polysciences, Inc.). 7 µm sections were cut by a RN2255 microtome and counter-stained with eosin. After mounted in Poly-Mount, the stained sections were photographed on a Provis AX-70 microscope equipped with a RETIGA EXi digital camera.

### Alkaline Phosphatase Staining

Embryos were fixed with 4% paraformaldehyde in PBS containing 0.5% Tween 20 (PBSTw) for 2 h at RT, washed with 50% methanol in PBS, and stored in 100% methanol for a week at −20°C. Samples were treated with pre-cooled 100% acetone for 30 min at −20°C, washed with PBSTw, and equilibrated with NTMT buffer (100 mM Tris (pH 9.5), 50 mM MgCl_2_, 100 mM NaCl, 0.1% Tween 20) at RT. Subsequently, samples were incubated with NBT/BCIP solution for 3–5 h at RT. Stained samples were dehydrated with a graded series of methanol and embedded in JB4 resin (Polysciences, Inc.). 7 µm sections were cut by a RN2255 microtome, and counter-stained with special eosin II. After being mounted in Poly-Mount (Polysciences, Inc.), the sections were imaged with a Provis AX-70 microscope equipped with a RETIGA EXi digital camera.

**Figure 6:**
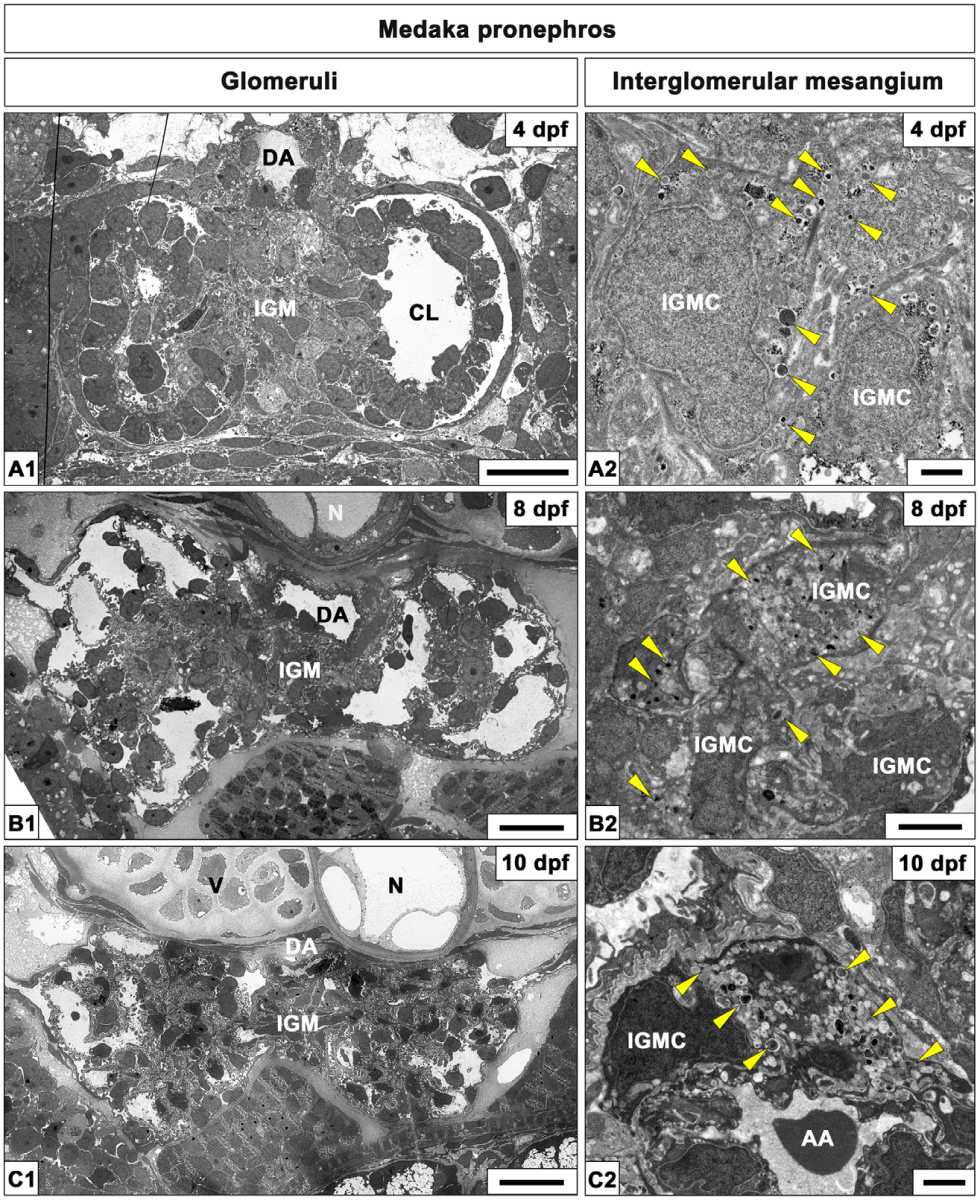
Interglomerular mesangium in medaka pronephric glomeruli. Ultrastructure of interglomerular mesangium of medaka pronephric glomeruli at 4 dpf (A1, A2), 8 dpf (B1, B2) and 10 dpf (C1, C2). Unlike zebrafish, the paired pronephric glomeruli are separated by interglomerular mesangium throughout medaka pronephric development (A1, B1, C1). The interglomerular mesangium is in close contact with the ventral surface of the dorsal aorta, which is located beneath the notochord or vertebra. The interglomerular mesangium consists of densely packed interglomerular mesangium cells and matrix. Most of the interglomerular mesangium cells contain granules which exhibit a variety of electron density (arrowheads in A2, B2, C2). Some interglomerular mesangium cells adhere to the afferent glomerular arteriole (AA) at 10 dpf (C2). CL, capillary lumen; DA, dorsal aorta; N, notochord; V, vertebra; IGM, interglomerular mesangium; IGMC, interglomerular mesangial cells; AA, afferent arteriole. Scale bars = 10 µm in A1, B1, C1; 1 µm in A2, B2, C2.

**Figure 7:**
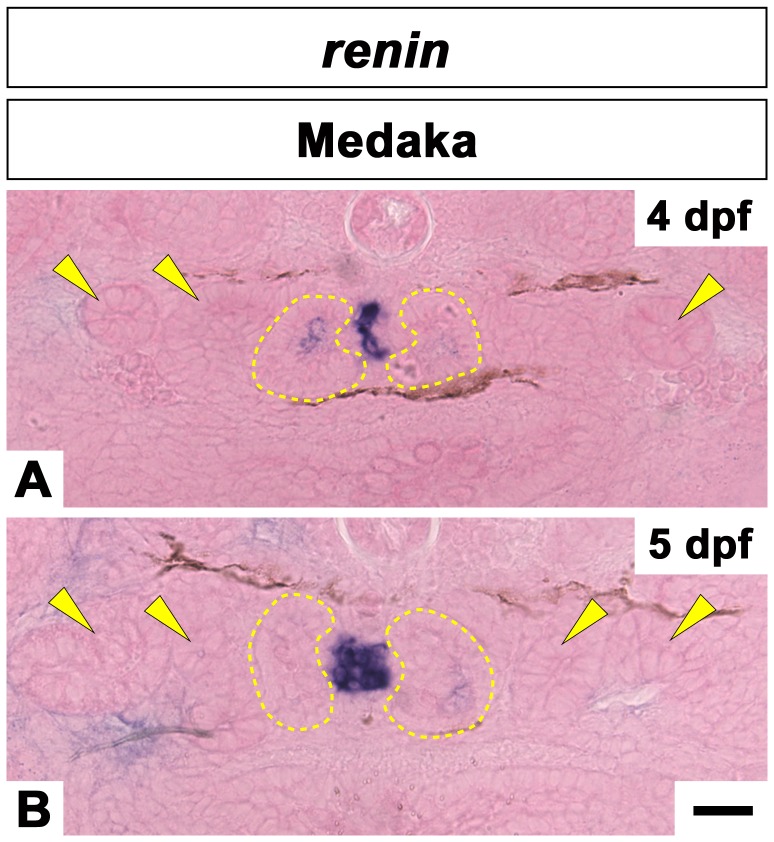
*renin* mRNA expression in the Interglomerular mesangial region in medaka embryos. *Renin* is expressed in the region between paired glomeruli, which are outlined by the yellow dotted lines, at 4 dpf (A) and 5 dpf (B). The location of the pronephric tubules are indicated by arrowheads. Scale bars = 10 µm.

### Periodic Acid-methenamine-silver (PAM) Staining

Embryos were fixed with 4% paraformaldehyde in PBS overnight at 4°C and dehydrated by graded series of methanol and embedded in JB4 resin. 7 µm sections were cut by a RN2255 microtome. PAM stain was performed by the use of an Accustain Silver Stain kit (Sigma-Aldrich). After being mounted in Poly-Mount (Polysciences, Inc.) the stained sections were imaged with a Provis AX-70 microscope equipped with a RETIGA EXi digital camera.

### Immunohistochemistry for Podocalyxin

Rabbit polyclonal anti-podocalyxin antibody (working dilution 1∶500) was raised against the glutathione S-transferase fusion protein with the entire cytoplasmic domain of rat podocalyxin [36]. Embryos were fixed with Dent’s fixative (20% DMSO in methanol) overnight at 4°C. Fixed samples were washed with PBS containing 0.5% Triton X-100 (PBSTx), blocked with blocking solution (PBS containing 0.5% Triton X-100, 10% normal goat serum, and 1% DMSO) and incubated overnight with the anti-podocalyxin antibodies diluted with incubation buffer (PBS containing 0.5% Triton X-100, 2% normal goat serum, and 1% DMSO). After washing with PBSTx, the samples were incubated for 2 h with Alexa-Fluor546-conjugated goat anti-rabbit IgG (H+L) (Jackson ImmunoResearch Laboratories) diluted with the incubation solution (working dilution 1∶200), dehydrated with a graded series of methanol, embedded in JB4 resin (Polysciences, Inc.), and cut into 5 µm sections. The sections were stained with DAPI (KPL), mounted in Fluorescent Mounting Media (KPL), and imaged with an FV-1000 confocal laser scanning microscope (Olympus).

**Figure 8:**
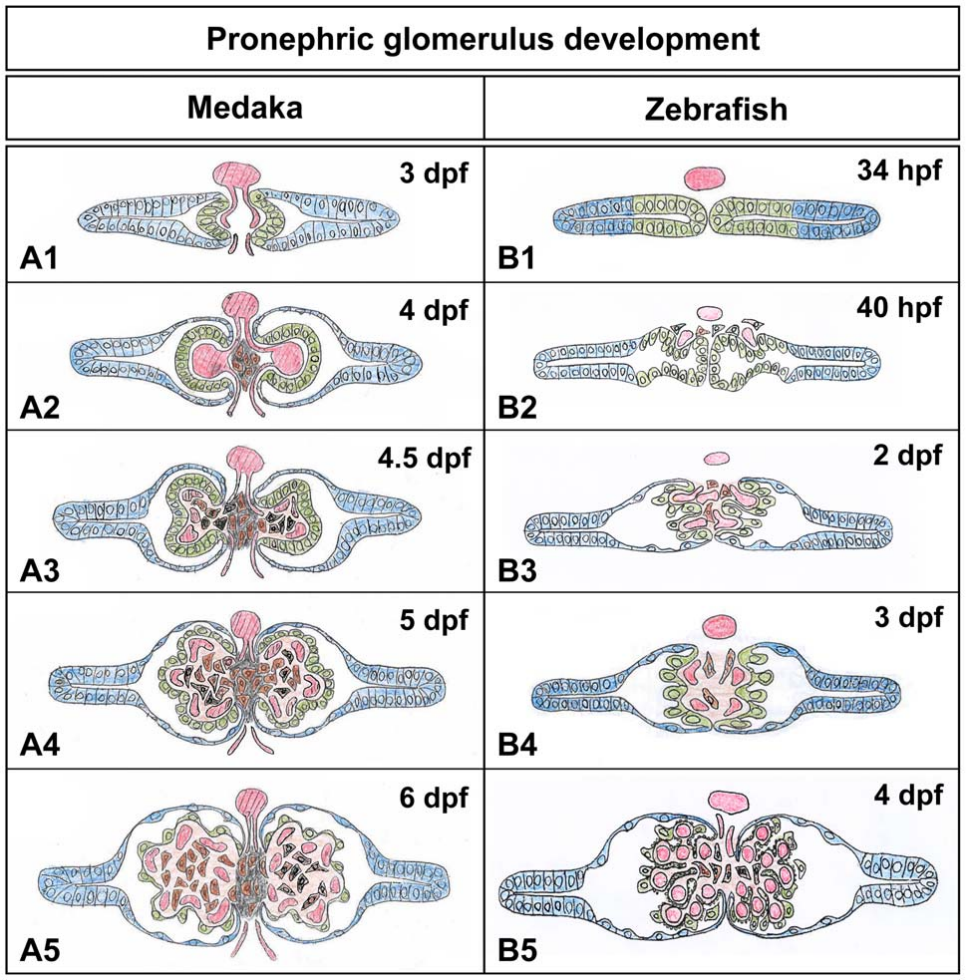
Summary of morphological processes during pronephric glomerulus development in medaka and zebrafish. Medaka glomerulus 3 dpf (A1), 4 dpf (A2), 4.5 dpf (A3), 5 dpf (A4) and 6 dpf (A5). Zebrafish glomerulus 34 hpf (B1), 40 hpf (B2), 2 dpf (B3), 3 dpf (B4) and 4 dpf (B5). Individual components of pronephric glomerulus are indicated by blue (tubular primordia and pronephric tubules), green (podocytes), red (glomerular capillary), brown (mesangial cell, interglomerular mesangial cells) and black (interglomerular mesangial matrix).

### Transmission Electron Microscopy

Embryos and larvae were immersed in histology fixative for overnight at 4°C. The fixed samples were processed by modified cold dehydration method. This method enabled detailed morphological observation of the extracellular matrices and cytoskeletons, as previously reported [37]. In brief, the samples were successively immersed in 0.4% OsO_4_ in 0.1 M PB for 1 h, 2% low molecular weight tannic acid (Electron Microscopy Sciences) in 0.05 M maleate buffer for 4 hours and 1% uranyl acetate in 0.05 M maleate buffer for 3 h. The samples were then dehydrated with a graded series of ethanol, and were embedded in Epoxy resin. Ultrathin silver-gold sections were produced with an ultra 45° diamond knife (Diatome), and were transferred to copper grids (50 mesh, Nisshin EM) which had been coated with Formvar membrane. The sections were then stained with uranyl acetate and lead citrate and observed with a JEM1230 transmission electron microscope (JEOL).

**Table 1:** Morphological types of pronephric glomeruli in teleost fishes.

Scientific name	Common name	Superorder	Order	Family	Reference
**Separated type**					
*Oryzias latipes*	Medaka	*Acanthopterygii*	*Beloniformes*	*Adrianichthyidae*	[31], Present study
*Dermogenys pusillus*	Halfbeak	*Acanthopterygii*	*Beloniformes*	*Hemiramphidae*	[48]
*Perca fluviatilis*	European perch	*Acanthopterygii*	*Perciformes*	*Percidae*	[52]
*Poecilia reticulata*	Guppy	*Acanthopterygii*	*Cyprinodontiformes*	*Poeciliidae*	[46]
*Scophthalmus maximus*	Turbot	*Acanthopterygii*	*Pleuronectiformes*	*Scophthalmidae*	[4]
*Elops hawaiensis*	Hawaiian ladyfish	*Elopomorpha*	*Elopiformes*	*Elopidae*	[48]
*Megalops cyprinoides*	Indo-Pacific tarpon	*Elopomorpha*	*Elopiformes*	*Megalopidae*	[48]
*Anguilla anguilla*	European eel	*Elopomorpha*	*Anguilliformes*	*Anguillidae*	[48]
**Fused type**					
*Danio rerio*	Zebrafish	*Ostariophysi*	*Cypriniformes*	*Cyprinidae*	[5], Present study
*Osteochilus hasseltii*	Hard-lipped barb	*Ostariophysi*	*Cypriniformes*	*Cyprinidae*	[48]
*Cirrhina mrigala*	Indian carp	*Ostariophysi*	*Cypriniformes*	*Cyprinidae*	[49]
*Chanos chanos*	Milkfish	*Ostariophysi*	*Gonorynchiformes*	*Chanidae*	[48]
*Salmo trutta*	Brown trout	*Protacanthopterygii*	*Salmoniformes*	*Salmonidae*	[52]
*Cyclothone sp.*	-	*Sternopterygii*	*Stomiiformes*	*Gonostomatidae*	[51]
*Gadus morhua*	Atlantic cod	*Paracanthopterygii*	*Gadiformes*	*Gadidae*	[47]
*Dicentrarchus labrax*	European sea bass	*Acanthopterygii*	*Perciformes*	*Moronidae*	[50]

* Lebistes reticulatus was adopted as scientific name of guppy in Agarwal and John (1988)[^46^].

## Results

### Overview of the Pronephric Glomerulus Development in Medaka and Zebrafish

In order to explore pronephric glomerulus development in medaka, we performed histological sections on embryos from 3 to 10 days post-fertilization (dpf). To compare the morphological differences between the medaka and zebrafish pronephric glomerulus, we also processed zebrafish embryos from 34 hours post-fertilization (hpf) to 4 dpf.

The pronephric nephron primordia for the glomerulus and tubule were already recognized as a pair of epithelial vesicles at the level of the pectoral fin buds in 3 dpf medaka (Fig. 1A1) and 34 hpf zebrafish (Fig. 1B1), as previously reported [5], [29], [38]. At 6 dpf or 4 dpf respectively, both fish species had formed a pronephric glomerulus and tubule, which was connected to the proximal end of the pronephric duct (Fig. 1A5, 1B5).

However, the morphological process of pronephric glomerulus formation was quite different between medaka and zebrafish. In medaka, the most medial portion of the vesicle invaginated into the lumen to form the glomerular primordium at 3 dpf (Fig. 1A1). The invaginated cells aligned themselves in a C-shaped epithelial layer of primitive podocytes at 4 dpf (Fig. 1A2). This cellular arrangement was similar to the corresponding structure in the mouse or rat S-shaped body; however, the C-shaped primordium of medaka also contained a balloon-like capillary, while the invaginating vasculature was not easily seen in the mouse or rat kidneys (Fig. 1C3, 1D3). At around 4.5 dpf, the balloon-like capillary had broken up into smaller capillaries interspersed by mesangial cells (Fig. 1A3). This initial association was followed by morphogenetic changes in the podocyte layer, which resulted in a concave-convex pattern. This arrangement was probably due to mechanical forces caused by the extensively developed glomerular capillary and mesangium and can also be seen in zebrafish, rat or mouse glomerulus at the capillary loop stage (Fig. 1A4, 1B4, 1C4, 1D4). At this time point, the urinary (Bowman’s) space between the parietal epithelium of Bowman’s capsule and the podocyte layer became apparent (Fig. 1A4, 1B4). Finally, by 6 to 7 dpf, the cuboidal appearance of the podocyte layer became more squamous and attained the light microscopic appearance of mature glomerulus morphology (Fig. 1A5) similar in the other three organisms (Fig. 1B5, 1C5, 1D5).

Importantly, in medaka, the paired glomeruli remained separated by a mass of interglomerular mesangium (IGM), which occupied the space between the two glomeruli and the dorsal aorta (Fig. 1A2–1A5). This continuous separation was in contrast to zebrafish, where the two glomerular primordia fused at the midline with glomerular capillaries by 2 dpf (Fig. 1B3).

### Development of the Individual Glomerulus Components

We next examined the development of the glomerulus by following the fate of the different cell populations that make up a functional glomerulus. To this end we used *wt1a* mRNA *in situ* hybridization and Podocalyxin immunohistochemistry to label podocytes, alkaline phosphatase staining for the endothelial cells in the glomerular capillary, PAM staining for the GBM and mesangial matrix and finally transmission electron microscopy for the underlying ultrastructure.

#### Podocytes

Glomerular podocytes in medaka and zebrafish pronephros were visualized by *wt1a* mRNA, which is predominantly expressed in this cell type throughout pronephric development [5], [38], [39]. In medaka, the invaginated portion of nephron primordium expressed *wt1a*, indicating that this portion is formed by primitive podocytes (Fig. 2A1). Similarly, the C-shaped epithelium at 4 to 4.5 dpf (Fig. 2A2, 2A3) and the podocyte epithelial layer that displayed a concave-convex appearance at 5 to 6 dpf (Fig. 2A4, 2A5) were *wt1a* positive. In zebrafish, the two entire flattened nephron primordia expressed *wt1a* at 34 hpf (Fig. 3A1). At the midline, *wt1a* expressing glomerular primordia were fused to form a single glomerulus by 2 dpf (Fig. 3A2), and *wt1a* expression in the podocytes is preserved at both 3 to 4 dpf (Fig. 3A3, 3A4).

Podocalyxin is primarily localized to the apical membrane of rat metanephric podocytes throughout its development [40], [41]. In other vertebrates (such as carp, bullfrog and newt) Podocalyxin is predominantly localized in the surface cell membrane of podocytes [7]. Therefore, we used Podocalyxin immunostaining to visualize pronephric podocyte development in medaka and zebrafish. At the S-shaped body stage of rat metanephric glomerulus development, apical membranes of the individual podocytes can be identified by the cap-shaped pattern of the Podocalyxin expression (arrowheads in Fig. 4A1). This pattern occurs because tight junctions of the developing podocytes are initially located within the upper third of the cell height. At early capillary loop stages, the junctions move closer to the GBM, causing Podocalyxin expression to assume a U-shaped pattern (arrowheads in Fig. 4A2). Finally, at the maturing glomerulus stage, Podocalyxin signal is found along the entire surface of the podocytes at the light-microscopic level (Fig. 4A3). A similar change of Podocalyxin expression occurs in both medaka and zebrafish. In medaka, at 3 dpf and 4 dpf Podocalyxin localizes to the individual podocytes in a U-shaped pattern (arrowheads in Fig. 4B1, 4B2) and is found in the entire surface of podocytes at 7 dpf (Fig. 4B3). In zebrafish, the cap-shaped pattern of Podocalyxin localization was observed at 34 hpf (arrowheads in Fig. 4C1), and expanded to encompass the entire surface of podocytes by 2 to 5 dpf (Fig. 4C2, 4C3).

In zebrafish, glomerular filtration starts at 40 hpf and the podocyte foot processes and the slit diaphragm are formed by 4 dpf [42]. In medaka, glomerular filtration is already observed at 10 dpf [43]. However, it is still unclear when glomerular filtration actually starts and when the foot processes and slit diaphragm are formed in medaka. Thus, we decided to further examine the cytoarchitecture of podocytes in medaka embryos from 4.5 to 10 dpf using transmission electron microscopy. At 4.5 dpf, podocytes formed a single columnar epithelium and neighboring podocytes were connected via intercellular junctions (arrowheads in Fig. 5A). These junctions were located in the middle of the cell. At 5 dpf, the intercellular junctions of podocytes were located in the vicinity of the GBM. Podocytes started to interdigitate at the cell periphery and formed irregularly-shaped processes (arrowheads in Fig. 5B). These processes were connected via the intercellular junctions. At 6 dpf, the podocyte cell body was detached from the GBM forming the subpodocyte space (asterisks in Fig. 5C), one of the characteristics of mature podocytes. Moreover, the irregularly-shaped processes had become flattened, but did not yet look like regular foot processes with a slit diaphragm (arrowheads in Fig. 5C). Those foot processes connected by slit diaphragm were initially formed in some capillary walls at 7 dpf (arrowheads in Fig. 5D) and were found in most capillary walls by 10 dpf (arrowheads in Fig. 5E).

#### Glomerular capillaries

The endothelial cells of the glomerular capillaries were detected by endogenous alkaline phosphatase [44]. In medaka, alkaline phosphatase-positive endothelial cells were already found in the invaginated portion of the nephron primordium (arrowheads in Fig. 2B1). Once the C-shaped podocyte epithelium was formed, a balloon-like capillary could be detected (Fig. 2B2). At 4.5 dpf, this capillary was divided into smaller capillaries interspersed by mesangium (Fig. 2B3). The glomerular capillaries then gradually grew (Fig. 2B4) and integrated into the mature glomerulus by 6 to 7 dpf (Fig. 2B5).

In zebrafish, the flattened nephron primordia formed in the proximity of the dorsal aorta. While the dorsal aorta exhibited alkaline phosphatase-positive endothelial cells (arrow in Fig. 3B1), glomerular capillaries could not be detected at 34 hpf. By 2 dpf the glomerular primordia were fused and individual capillaries could be detected (Fig. 3B2). By 3 to 4 dpf, the glomerular capillaries were integrated with the pronephric glomerulus (Fig. 3B3, 3B4) as described previously [42].

#### Mesangium and GBM

In zebrafish, a pair of pronephric glomerulus primordium fused to form a glomerulus at the midline (Fig. 1B1-B5) [5]. In contrast, in medaka, the IGM is positioned between the two pronephric glomerulus primordia that remain as two separated glomeruli (Fig.1A1-A5). To identify the characteristics of mesangium, IGM and GBM, we further investigated these structures by PAM staining to visualize reticular fibers and basement membranes and by transmission electron microscopy to visualize ultrastructure.

In medaka, the invaginated portion of the basement membrane primordium was visible by PAM staining (arrowheads in Fig. 2C1) at 3 dpf. By 4.5 dpf the GBM displayed a tortuous appearance (Fig. 2C3), which became progressively more complex with the development of the glomerular capillaries at the later time points (Fig. 2C4, 2C5). In zebrafish, the GBM of the paired glomerular primordia were in contact with each other at the midline at 34 hpf (Fig. 3C1). Once the glomerular primordia and capillaries fused at 2 dpf, the GBM displayed a tortuous appearance (Fig. 3C2, 3C3). In mature metanephric glomeruli of rodents and humans, PAM staining is also used to visualize the mesangial matrix [45]. However, we did not detect mesangial matrix by PAM staining in the pronephric glomeruli of either fish species (Figs. 2C5, 3C4). In zebrafish, the pronephric glomeruli are fused but IGM was not formed and PAM staining was only observed in the basement membrane of the glomerulus. Conversely, in medaka, from 4 until 6 dpf, the IGM was PAM-positive, and amorphous in appearance (arrowheads in Fig. 2C2–2C5).

Next we used transmission electron microscopy to provide a more detailed view of interglomerular mesangial cells (IGMCs) in medaka (Fig. 6). At 4 to 4.5 dpf, most of the IGMCs contained small granules that were various in electron density (arrowheads in Fig. 6A2). The number and density of these granules increased with development (Fig. 6B2, 6C2). Some IGMCs adhered to the afferent arteriole at 10 dpf, which was reminiscent of renin-producing (juxtaglomerular) cells seen in afferent arteriole of mammals (Fig. 6C2). In 4 and 5 dpf medaka embryos, *renin* mRNA expression was detected by *in situ* hybridization at the interglomerular regions (Fig. 7). Based on these data, we supposed that IGMCs expressed *renin* and their cytoplasmic granules contained Renin protein.

## Discussion

Medaka and zebrafish are two fish species that are widely used for both genetic and embryological studies. Here we defined and compared the specific pronephric morphological processes that lead to pronephric glomerulus development (summarized in Figs. 8, S1). The main characteristics of pronephric glomerulus development in medaka were as follows: (1) The glomerular primordium of the medaka pronephros exhibited a C-shaped epithelial layer, unlike in zebrafish. (2) The C-shaped primordium contained a characteristic balloon-like capillary, which later divided into several smaller capillaries. (3) A pair of pronephric glomeruli were fused at the midline to form a glomerulus in zebrafish, but remained independent of each other in medaka due to the interposition of the IGM between them. (4) The IGMCs possessed numerous cytoplasmic granules throughout pronephric development, which were highly likely to contain Renin protein.

In vertebrates, pronephric glomeruli protrude directly into the coelomic cavity (the so-called external glomerulus), and the proximal end of the pronephric tubule is opened into the coelomic cavity *via* nephrostome(s) [1]. However, in teleost fishes including medaka and zebrafish, the pronephric glomeruli are encapsulated by the parietal epithelium of Bowman’s capsule to form a renal corpuscle, which is connected to the pronephric tubule, as seen in the mesonephric and metanephric glomerulus.

The morphological features and development of pronephric glomerulus have been examined in a variety of teleost species, as summarized in Table 1 and Fig. S2 [4], [5], [31], [46]–[53]. Some of these species such as zebrafish exhibit a single pronephric glomerulus formed by the fusion of two original glomeruli (fused type). Others such as medaka have two pronephric glomeruli that remain separated from each other (separated type). The separated type is also found in some fishes of the superorder *Elopomorpha* (ladyfish, tarpon, and eel) [48], which is regarded as one of the most primitive groups among teleost fishes [54]. Therefore, it is likely that the separated type of pronephric glomeruli is an original form in teleost, and that the fused type is a specialized form in some taxa.

In the metanephric glomerulus of mammals, extraglomerular mesangium (EGM), one of the components of juxtaglomerular apparatus, occupies the space bounded by the macula densa of distal tubule and glomerular arterioles at the glomerular hilum (vascular pole) [55]. Both the IGM and EGM are situated at the glomerular hilum, but their biological functions are presumably quite different. The extraglomerular mesangial cells contain abundant actin filament bundles, and act in the mechanical protection of glomerular hilum to prevent expansion of the hilum in response to higher intraglomerular pressure [55], [56]. On the other hand, in the pronephric glomerulus of medaka, IGMCs display the morphological features of secretory cells and are reminiscent of the renin producing (juxtaglomerular) cells found in the afferent arteriole wall of the mammalian metanephric glomeruli. Since *renin* mRNA-expressing cell were detected by *in situ* hybridization in the IGMCs, we will need to examine whether the granules contain Renin protein as we predict. In mice, (pro)renin protein and its receptor are thought to play a role in structural integrity and function of podocytes during pre- and postnatal growth [57]. Additional studies are needed to understand whether (pro)renin and its receptor are involved in the formation of the glomerulus in zebrafish and medaka development.

Mesonephric glomeruli start to form by 4–5 dpf in medaka and nephrogenesis continues in juvenile fish [31], [58]. Three different stages of nephrogenesis can be distinguished in the mesonephros, (1) mesenchymal condensation, (2) formation of nephrogenic body, and (3) maturation of nephron [31], [59]. The nephrogenic body is an epithelial vesicular structure in which the proximal end invaginates into the lumen, as seen in the pronephric nephron primordium. However, it remains unclear whether the nephrogenic body in the mesonephros develops in the same manner as seen in the pronephric nephron primordium.

The balloon-like glomerular capillary that was formed within the C-shaped developing glomerulus in the medaka pronephros is similar to the sinusoidal capillary that has been reported for the developing mesonephric glomerulus of bullfrog and *Xenopus laevis* [60], [61]. The sinusoidal capillary in these frogs is subsequently remodeled into a glomerular tuft, as seen in the medaka. The vascular resin casts of the sinusoidal capillary display several tiny holes (tunnels), which are known to be a characteristic morphological feature found in intussusceptive capillary remodeling [62]–[66]. In this type of capillary remodeling, the tiny hole and a transluminal tissue pillar in the hole become enlarged in diameter to split the existing capillary into two small daughter ones, thereby increasing the overall capillary density. It is reasonable to propose that the sinusoidal glomerular capillary is split into smaller capillaries by intussusceptive remodeling, and it is also likely that the balloon-like capillary in medaka is remodeled in the same way. Intussusceptive capillary remodeling is involved in the formation of complex capillary networks both in development and disease [62]–[66]. For example, in rat Thy-1.1 nephritis, a model for human proliferative glomerulonephritis, mesangial cells are largely destroyed resulting in a simplified glomerular capillary [67]. Importantly, intussusceptive capillary remodeling and the mesangial cells contribute to restoration of the glomerular capillary tufts injured in this nephritis [68]–[70]. Therefore, mesangial cells may also be involved in the process of intussuceptive capillary remodeling during pronephric glomerulus development in medaka.

There are several advantages in studying glomerular development in the medaka pronephric glomerulus compared to zebrafish. For example, the morphological process of podocyte differentiation in medaka is more similar to mammals. In particular, the glomerular primordium of the medaka pronephros exhibits a C-shaped epithelial layer of primitive podocytes, which is similar to that of mammalian S-shaped body. We predict that the morphological processes involved in podocyte development will show significant parallels between medaka and mammals.

In conclusion, we have thoroughly described the developmental processes of pronephric glomerulogenesis in medaka, demonstrating that stark differences exist between medaka and zebrafish. This study provides the groundwork for future phenotypic analyses of pronephric glomerular defects in medaka mutants, which should elucidate general aspects of glomerulus function and potentially provide insight into human glomerular diseases.

## Supporting Information

Figure S1 **Time course of pronephric glomerular development in medaka and zebrafish.**
(TIF)

Figure S2 **Phylogeny and morphological types of pronephric glomeruli in teleost fishes.** Phylogeny of the teleost fishes listed in Table 1.
(TIF)
