# Supplementary figures and images for: A Comparative Analysis of Glomerulus Development in the Pronephros of Medaka and Zebrafish
Source: PLoS One. 2012 Sep 18;7(9):e45286. doi: 10.1371/journal.pone.0045286 (PMC3445478; doi:10.1371/journal.pone.0045286)

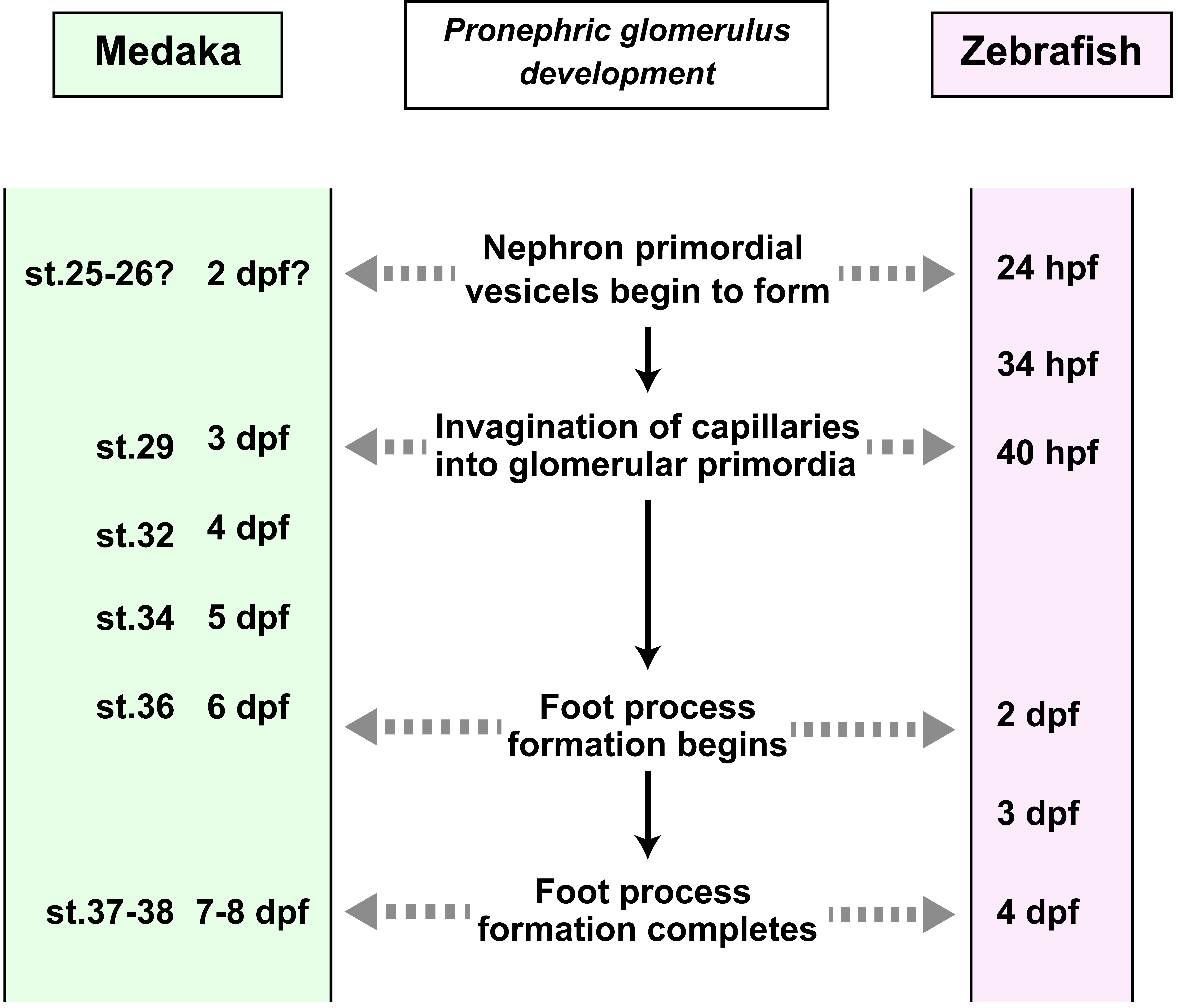

Supplement: Figure S1 — Time course of pronephric glomerular development in medaka and zebrafish. (TIF) [file pone.0045286.s001.tif]
